# Estrogen deficiency reduces the dentinogenic capacity of rat lower incisors

**DOI:** 10.1007/s10735-013-9533-4

**Published:** 2013-08-22

**Authors:** Tao Xu, Ming Yan, Yanping Wang, Zhanwei Wang, Lizhe Xie, Chunbo Tang, Guangdong Zhang, Jinhua Yu

**Affiliations:** 1 Institute of Stomatology, Nanjing Medical University, 140 Hanzhong Road, Nanjing, 210029 Jiangsu China; 2 Department of Stomatology, Nanjing Governmental Hospital, 116 Chengxian Road, Nanjing, 210018 Jiangsu China; 3 Department of Endodontics, School of Stomatology, Nanjing Medical University, 136 Hanzhong Road, Nanjing, 210029 Jiangsu China; 4 Department of Endodontics, Suzhou Stomatological Hospital, 1505 Renmin Road, Suzhou, 215005 Jiangsu China; 5 Cancer Biology Program, University of Hawaii Cancer Center, 701 Ilalo Street, Honolulu, HI 96813 USA; 6 Department of Prosthodontics, School of Stomatology, Nanjing Medical University, 136 Hanzhong Road, Nanjing, 210029 Jiangsu China

**Keywords:** Ovariectomy, Estrogen, Tooth, Dentinogenesis, Mineralization

## Abstract

Endogenous estrogen deficiency usually causes the systemic osteoporosis including the jaw bones. However, it remains unclear whether estrogen deficiency can affect the tooth mineralization. In this study, the classical osteoporotic rat model was established via the ovariectomy, which was subsequently confirmed by the detection of serum estradiol levels and body weights. The mineralization-related assays were performed to observe the potential changes in mineralized tissues of rat lower incisors. The clinical crown length, compressive strength, radiodensity, and calcium content in the ovariectomy group (OVX) were significantly downregulated (*P* < 0.01), as compared with the sham operation group (Sham). Histological results revealed that OVX incisors presented the thinner predentin structures than Sham incisors. Immunohistochemical staining and western blot assay further demonstrated that the odonto/osteoblast specific proteins (e.g., dentin sialoprotein, runt-related transcription factor 2, osterix, and osteocalcin) in the dentin–pulp complex of OVX incisors were significantly decreased in comparison with Sham counterparts. Together, estrogen deficiency reduces the dentinogenic capacity and calcium deposition in rat incisors, indicating that estrogen plays an important role in the dentinogenesis.

## Introduction

Estrogen deficiency-induced postmenopausal osteoporosis is a kind of world-wide systemic disease, which is characterized by the bone loss, skeletal fragility and even bone fractures in the middle-aged and older women (Johnell and Kanis 2006; Khosla et al. 2011; Lyles et al. 1993; Nevitt et al. 1998; Orwoll and Nelson 1999; Reginster and Burlet 2006; Rossini et al. 2013). Some scholars have proved the positive correlation between systemic estrogen deficiency and vertebrae/long bone fractures (Ejiri et al. 2008; Ettinger et al. 1992; Lyles et al. 1993; Nevitt et al. 1998), which subsequently affects the quality of life and even cause mortal diseases. In dentistry, the influence of estrogen deficiency on the jaw bones has drawn the attention of researchers and clinicians (Ejiri et al. 2008). Many studies have showed that the estrogen deficiency can bring about some changes in jaw bones which are related to the skeletal bone abnormalities (Corten et al. 1993; Nakamoto et al. 2003; Taguchi et al. 2007a, b). Moreover, patients with a history of osteoporotic fractures tend to have an increased mandibular bone loss with the appearance of trabecular structural modifications (Bollen et al. 2000; White and Rudolph 1999). To date, little knowledge is available about the relationship between estrogen deficiency and tooth metabolism.

As an animal model, the ovariectomized (OVX) rat has been widely used in the osteoporosis research, as it has been validated to cause the estrogen deficiency and present similar post-menopausal bone loss in adult human beings suffering from the early-stage osteoporosis (Kalu 1991; Namkung-Matthai et al. 2001). Some studies have demonstrated that the ovariectomy-induced bone loss is attributed to the increase of bone formation in addition to its enhancement in bone resorption (Nagao et al. 2011). Moreover, OVX can reduce the mineralization density of alveolar bone, affect the healing process of bone socket following the tooth extraction, and increase the turnover of alveolar bone in the healed extraction socket in rat incisors (Rawlinson et al. 2009; Shirai et al. 2002; Shoji et al. 2011). Yet, whether OVX can exert the similar influence on the mineralization of rat incisors remains unclear.

Earlier studies have showed that exogenous estrogen causes the alterations in the ground substance of dentin which are similar to the changes observed in bone (Bernick and Ershoff 1963; Zussman 1965), while estrogen deficiency brings about the decrease of copper content in rat teeth (Rahnama 2002). Our previous work has proved that estrogen deficiency can down-regulate the odonto/osteogenic differentiation of dental pulp stem cells (DPSCs) by activation of NF-κB signaling (Wang et al. 2013). Since DPSCs are pivotal to the regeneration of tooth and dental–pulp complex (Yan et al. 2011; Yu et al. 2006; Yu et al. 2008), estrogen deficiency should have some impact on the tooth formation and mineralization. In this study, we hypothesized that OVX-induced estrogen deficiency can exert some negative effects on the mineralization and dentinogenesis of rat incisors. For this purpose, ovariectomy was performed in adult female rats to create the model of estrogen deficiency and the mineralization-related features of lower incisors were evaluated.

## Materials and methods

### Establishment of estrogen-deficient animal model

48-week-old female Sprague–Dawley rats (Experimental Animal Center of Nanjing Medical University) were randomly divided into ovariectomized (OVX) and sham (Sham) operation groups. Firstly, the rats were anesthetized and the fur on their back was shaved. Secondly, under sterile conditions, a dorsoventral incision was made on each side of the paralumbar fossa. The ovary was removed through the incision, and the remaining fat (with the ovary attached in Sham group and without the ovary in OVX group) was returned to the abdominal cavity. Thirdly, abdominal membrane, muscle and skin were sutured respectively. Finally, all rats were given free diet and drink. The animals were sacrificed 1 month post operation (Kaczmarczyk-Sedlak et al. 2013; Wang et al. 2013). The animals were treated according to the animal experimental guideline approved by the Animal Welfare and Research Committee of Nanjing Medical University.

### Detection of serum estradiol and body weight

After the rat was euthanized by an overdose of pentobarbital, 1.5 mL of blood was taken via cardiac puncture, centrifuged for 5 min, and the serum estradiol levels were detected by the immunochemiluminescent assay with UniCel DxI800 Immunoassay System (Beckman Coulter Inc.). Body weights in two groups were respectively recorded at 0 or 1 month after surgery (Kaczmarczyk-Sedlak et al. 2013; Wang et al. 2013). Ten rats were used in this experiment and data were described as the mean ± SD.

### Macromorphology and radiodensity of incisors

One month after operation, mandibles containing lower incisors were isolated. The length and shape of clinical crowns in both groups were recorded and evaluated. After thoroughly removing the soft tissues around the incisors, CCX digital system (SATELEC, X-MIND, France) was used to detect the radiodensities of incisors at 70 kV (8 mA, 0.08 s) with the working distance of 20 cm. Radiodensity levels of these films were represented as mean optical density (MOD) values which were calculated by Image-Pro Plus 5.0 software (de Melo Mde et al. 2013; Martins et al. 2005). Briefly, after intensity calibration by the software, MOD values were described as the ratio of integral optical density to the area, which is proportional to the radiodensity of lower incisors. Ten mandibles from different rats in each group were used for in vitro analysis of radiodensity.

### Compressive strength measurement

**Fig. 1:**
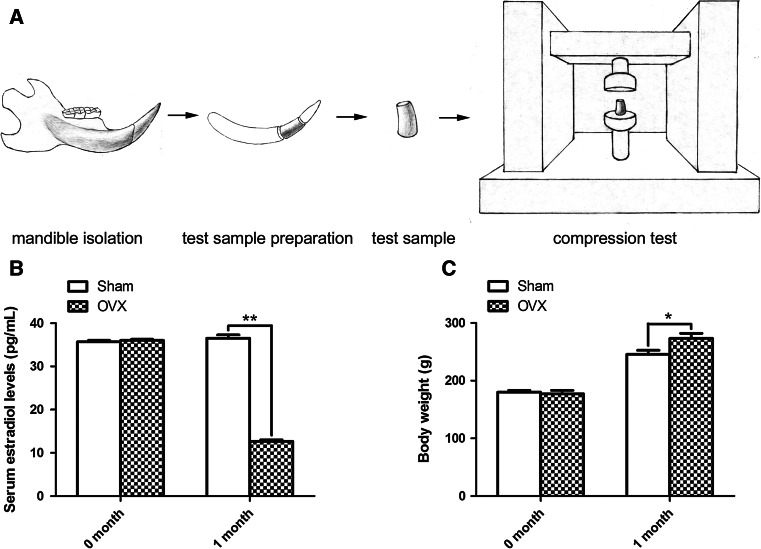
Schematic diagram of compression test and establishment of ovariectomized rat model. **a** Schematic diagram of the compression tests for rat incisors. Lower incisor was isolated from the rat mandible and test sample was made of the lower incisor. Then, the compression test was performed using Electronic Universal Testing Machine. **b** One month after the ovariectomy, the serum estradiol levels in OVX rats were significantly decreased, as compared with Sham rats. **c** One month following the ovariectomy, OVX rats were obviously heavier than Sham rats. *P < 0.05, **P < 0.01

The lower incisors were isolated from rat mandibles, and the forepart 2 mm of lower incisors was discarded. Then, 2 mm of the remaining incisor crown was isolated from the anterior part as the test sample. Its shape is like the frustum of a cone. At last, the compressive strength value of each sample was detected by Electronic Universal Testing Machine (INSTRON 3365, USA). Figure 1a shows the schematic diagram of the compression test in which ten lower incisor samples from different rats were used in each group.

### Alizarin red staining and calcium detection

Ten lower incisors from different rats in each group were isolated and crushed. 0.12 g debris of each incisor was thoroughly homogenized in the deionized water and centrifuged to discard the supernatant. Each sample was stained with 2 % alizarin red (pH = 4.2) for 2 min, and then rinsed by deionized water for 3 times. Then, calcium contents were quantitatively analyzed by cetylpyridinium chloride (CPC) assay as described before (Fan et al. 2009). 4 mL 10 % CPC was added into these samples, followed by vortexing for 2 h and centrifugation. The acquired supernatant was diluted ten times and analyzed by ELISA Reader (490 nm). The final calcium concentrations were normalized to total protein content and described as ng per mg of protein.

### Histological and immunohistochemical (IHC) staining

Ten incisor samples from different rats in each group were fixed in 4 % polyoxymethylene for 24 h and processed for hematoxylin and eosin (H&E) staining. Immunohistochemical analyses of these incisors were performed by the streptavidin–biotin complex (SABC) method according to the manufacturer’s recommended protocol. Briefly, tissue sections (5 μm) from representative paraffin blocks were deparaffinized in xylene and rehydrated through gradient ethanol solutions. For the antigen-epitope retrieval, the sections were processed by the conventional microwave heating in 0.01 M citrate buffer (0.01 M sodium citrate and 0.01 M citric acid, pH = 6.0) for 5 min. Sections were treated with 100 μL 3 % H_2_O_2_ to suppress the endogenous peroxidase activity for 10 min at room temperature. Then, sections were blocked by 5 % normal goat serum for 1 h and then incubated with the primary antibodies (DSP, 1:200, Santa Cruz; RUNX2, 1:100, Bioworld; OSX, 1:100, Abcam; OCN, 1:100, Abcam) overnight at 4 °C. Incubation with PBS instead of primary antibodies served as the negative controls. Sections were rinsed with PBST and incubated with biotinylated secondary antibodies for 45 min at room temperature. Finally, sections were washed three times with PBST, incubated with SABC for 30 min, and stained with 100 μL DAB solution. When the brown color was detected, slides were counterstained with hematoxylin for 1 min and observed under the light microscope. This experiment was repeated three times.

### Western blot analysis

Ten lower incisors were carefully isolated from the mandibles after 1 month of operation, and thoroughly homogenized in RIPA buffer (Beyotime, China) containing 1 mM phenylmethylsulfonyl fluoride (PMSF). The debris of each sample was eliminated by centrifugation at 12,000 rpm for 10 min. Protein concentrations were determined via Bio-Rad protein assay kit (Pierce, Rockford, IL). 40 μg proteins per lane were loaded on a 10 % SDS-polyacrylamide gel for electrophoresis, and then transferred onto PVDF membranes (Millipore Co. Bedford, MA, USA) at 300 mA for 1 h in a blotting apparatus (Bio-RAD, CA, USA). Membranes were blocked at room temperature for 2 h with blocking solution (5 % w/v skim milk, 0.01 mol/L PBS, 0.1 % Tween-20), and subsequently incubated overnight at 4 °C with primary polyclonal antibodies (DSP, 1:1,000, Santa Cruz, Delaware, CA; RUNX2, 1:1,000, Bioworld; OSX, 1:1,000, Abcam; OCN, 1:1,000, Abcam; β-actin, 1:1,000, Abgent). Then, the membranes were rinsed with PBST (0.1 % Tween-20 in 0.01 mol/L PBS), incubated with the appropriate horseradish peroxidase conjugated secondary antibodies at 1:10,000 (Boster) at room temperature for additional 1 h, visualized by SuperSignal West Pico Chemiluminescent Substrate (Thermo, Rockford, USA), and exposed to Kodak X-ray films. β-actin served as the internal control. This experiment was performed in triplicate.

### Statistics

The quantitative results were expressed as the mean ± SD. Independent samples *t* test were performed with SPSS-Windows v.12.0 software. *P* values less than 0.05 were considered statistically significant.

## Results

### Effects of estrogen deficiency on estradiol levels and body weights

OVX and Sham rats were sacrificed at 1 month after surgery, their estradiol levels and body weights were respectively measured. As presented in Fig. 1b, c, there was no statistically significant difference (*P* > 0.05) between two groups in estradiol levels and body weights at 0 month. One month after surgery, the estradiol levels in OVX group were significantly lower than Sham group (Fig. 1b, *P* < 0.01) while OVX rats were significantly heavier than Sham rats (Fig. 1c, *P* < 0.01), indicating the successful establishment of estrogen-deficiency animal model.

### Effects of estrogen deficiency on compressive strength and mineralization of rat incisors

**Fig. 2:**
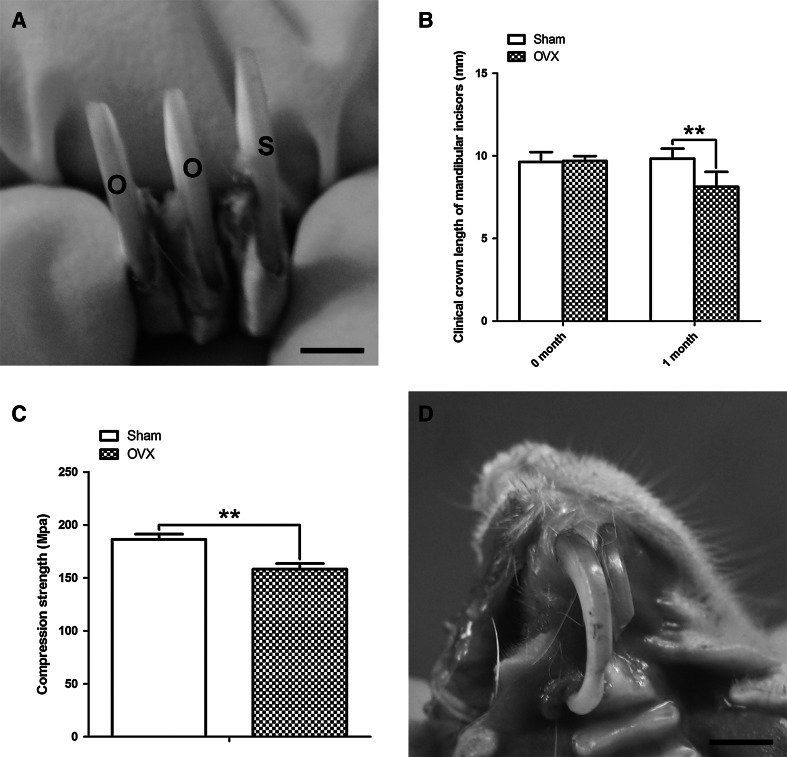
Changes in crown length and strength of ovariectomized incisors. **a**, **b** One month after the ovariectomy, the clinical crown height in OVX incisors (O) was significantly shorter than Sham ones (S). **c** As compared with Sham incisors, the compressive strength in OVX group was significantly decreased. **d** Shape abnormality (bending and broken) in the upper incisors of OVX rats. *Scale bars* = 2 cm. **P < 0.01

The clinical crowns of lower incisors in OVX group were shorter than those in Sham group (Fig. 2a, b, *P* < 0.01) at 1 month after surgery. Under the Electronic Universal Testing Machine, the compressive strengths in OVX samples were higher than those in Sham group (Fig. 2c, *P* < 0.01) at 1 month post-operation. Moreover, some upper incisors became abnormal in shape and even fractured in OVX group (Fig. 2d), as compared with Sham group.

**Fig. 3:**
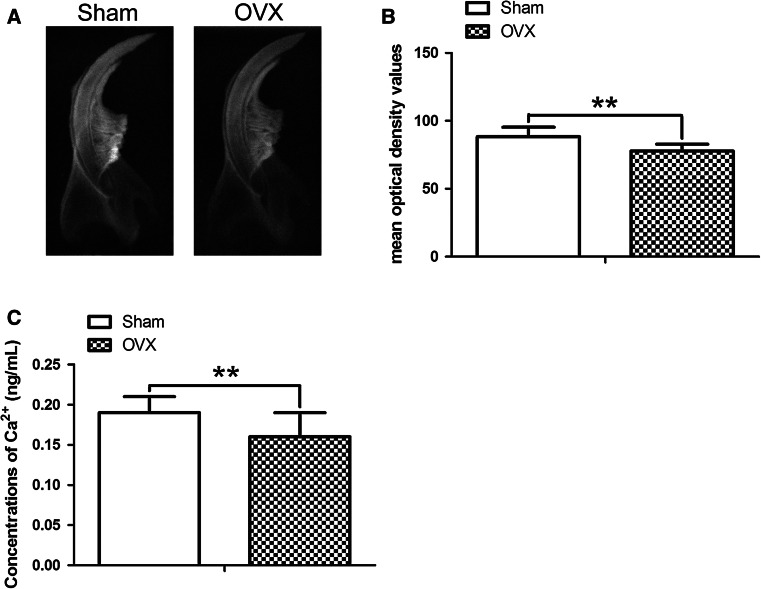
Changes in radiograph and calcium content of ovariectomized incisors. **a** The radiographs of Sham and OVX incisors/mandibles. **b** The MOD values of X-ray films in OVX group were significantly lower than Sham group by the analysis of Image-Pro Plus 5.0 software. **c** The calcium concentrations in OVX incisors were less than those in Sham incisors. **P < 0.01

X-ray photography and grayscale value analysis by Image-Pro Plus 5.0 software demonstrated that the radiodensity of lower incisors and mandibles in Sham group was higher than that in OVX group (Fig. 3a), in which Sham group exhibited the higher grayscale values than OVX group (Fig. 3b, *P* < 0.01). Moreover, the concentrations of calcium ion in lower incisors decreased in OVX incisors as compared with Sham incisors (Fig. 3c, *P* < 0.01).

### Effects of estrogen deficiency on the predentin thickness and protein expression of rat incisors

**Fig. 4:**
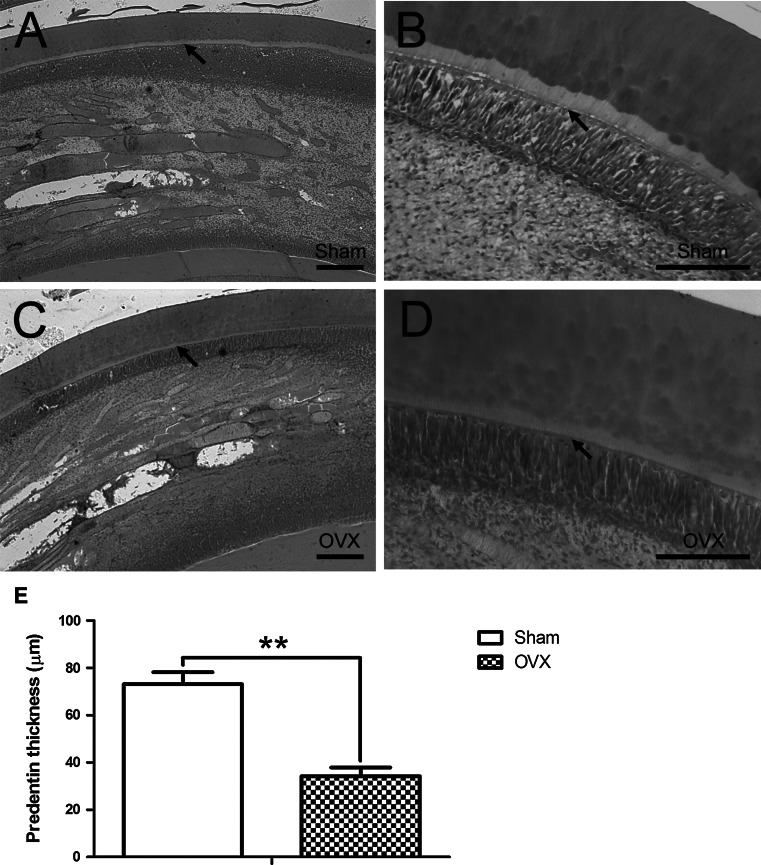
Changes in predentin thickness of ovariectomized incisors. Longitudinal sections of Sham incisors (**a**, **b**) and OVX incisors (**c**, **d**) by H&E staining. Sham incisors presented the thicker predentin structures (*arrows*) than OVX incisors. **e** Average thickness of predentin structures respectively in Sham and OVX groups. *Scale bars* = 50 μm

**Fig. 5:**
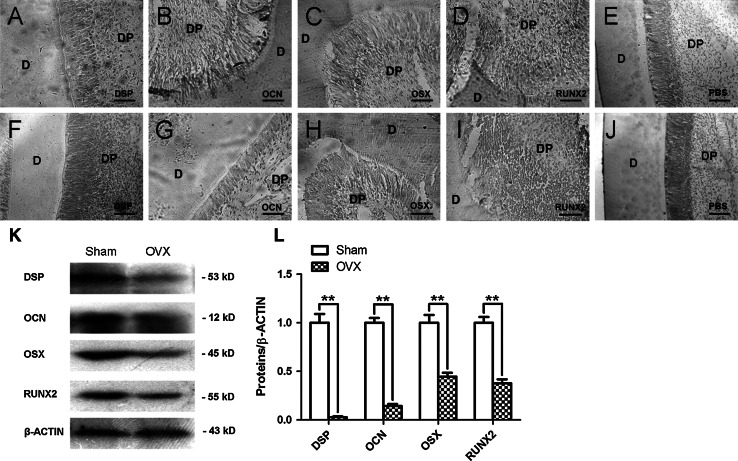
Changes in mineralization-related proteins of ovariectomized incisors. (**a**–**j**) Sham group presented the stronger immunostaining for DSP (**a**), OCN (**b**), OSX (**c**) and RUNX2 (**d**) in the dentin–pulp complex than OVX group (**f**–**i**). **k** Protein expressions of DSP, OCN, OSX and RUNX2 in the lower incisors. β-actin was used as a control. **l** *Grayscale* analysis of Fig. 5k by Image-Pro Plus 5.0 software. *Scale bars* = 50 μm. *D* dentin, *DP* dental pulp

Histological assay revealed that the predentin structures in Sham incisors (Fig. 4a, b and e) were thicker than those in OVX incisors (Fig. 4c, d and e). Immunohistochemical staining demonstrated that the protein expressions of DSP, OCN, OSX and RUNX2 in predentin structures were stronger in Sham incisors (Fig. 5a–d) than those in OVX incisors (Fig. 5f–i). Moreover, the odontoblastic layer and cell-rich zone in Sham incisors showed a stronger staining for DSP than those in OVX incisors. Interestingly, the staining for DSP, OCN, OSX and RUNX2 became stronger in the cell-rich zone of Sham incisors as compared with OVX incisors. No significant difference between two groups was detected in the expression of mineralization-related proteins in dentin structures. Western blot assay further confirmed that the protein expressions of DSP, OCN, OSX and RUNX2 were significantly weaker (*P* < 0.01) in OVX group as compared with Sham group (Fig. 5k and l).

## Discussion

Estrogen deficiency has been widely reported to cause the osteoporosis in vertebrae, long bones as well as jaw bones, and even bring about the bone fracture in old women due to the loss of calcium contents (Ejiri et al. 2008; Ettinger et al. 1992; Johnson et al. 1997; Khosla et al. 2011; Lyles et al. 1993; Nevitt et al. 1998; Orwoll and Nelson 1999; Rossini et al. 2013). However, it remains unclear whether estrogen deficiency can result in the dentin deterioration and altered protein expression in dental tissues. This study established the OVX rat model and examined the changes of rat incisors in case of estrogen deficiency. The cumulative findings revealed that the abrasion in OVX incisors was accelerated because of the decreased mineralization ability, as indicated by the shorter clinical crowns, reduced compressive strength, decreased radiodensity, and less calcium contents in OVX incisors. This dental pathosis may be attributed to the faster calcium loss, dentin deterioration, declined inorganic mineralization and increased organic compounds in OVX incisors, which is similar to the changes appeared in bone structures suffering from estrogen deficiency (Brennan et al. 2011; Brennan et al. 2012; Park et al. 2008).

Moreover, OVX incisors presented the thinner predentin structures than Sham incisors, indicating that estrogen deficiency can impair the dentinogenic capacity of dentin–pulp complex in rat incisors. Various studies have proved that predentin can provide a mechanical supporting function for the pulp tissue and the thickness of predentin layer reflects the function of dentinogenic activity in human teeth. At the growing end next to the apex of human developing teeth where dentinogenesis is most active, predentin exhibits its greatest thickness, whereas at the coronal region where primary dentin has been completely formed, predentin width is dramatically reduced to a mean value of 14.8 micron (Couve 1987).

We also gained insights into the role of some mineralization related proteins, such as DSP, OCN, OSX and RUNX2. DSP has great significance to odontoblast differentiation and dentin mineralization. As a specific marker of odontoblast, DSP can adjust the shape, size and growth rate of hydroxyapatite, and subsequently affect the dentinogenesis (Lee et al. 2009; McKnight et al. 2008; Rajpar et al. 2002). OCN is the late-stage marker of osteoblast differentiation. As a kind of transcription factors of osteoblast lineages, RUNX2 plays an important role in the formation and differentiation of osteoblasts, and even can be regarded as the switch of cell differentiation (Ducy et al. 1997). Interference of *RUNX*2 gene will inhibit the generation of mineralization nodules as well as the expression of other genes controlling bone formation (Komori et al. 1997; Mundlos et al. 1997; Otto et al. 1997). Moreover, RUNX2 is very important to the tooth development, and affects the formation and mineralization of dentin structures (Kobayashi et al. 2006). Mutation of *runx*2 gene will lead to the down-regulation of *dspp* gene (Chen and Messer 2002). OSX is a downstream target of RUNX2, which represents another key regulator of osteoblast differentiation. RUNX2 regulates the osteoblast differentiation via OSX and OSX-knockout mice have no bone formation (Nakashima et al. 2002). In this study, OVX group presented the weakest expression of several odonto/osteogenic proteins (e.g., DSP, OCN, OSX and RUNX2) in the dentin–pulp complex than Sham incisors, indicating that estrogen deficiency downregulated the dentinogenesis, and finally caused the declined mineralization and weakened strength in OVX incisors. Since cell-rich zone mainly contains pulp fibroblasts and dental pulp stem cells (d’Aquino et al. 2009; Tirino et al. 2012), the upregulation of these odonto/osteogenic proteins in it may imply that the cell-rich zone in Sham incisors was in an active state of differentiation in comparison with OVX incisors.

In conclusion, lack of estrogen has a negative influence on the dentin formation, calcium deposition and compressive strength, indicating that estrogen can regulate the dentinogenesis and calcium deposition of rat incisors. From the clinical point of view, long-term estrogen deficiency may not only lead to the osteoporosis but also result in the impaired mineralization and decreased regeneration capacity of dentin–pulp complex. Moreover, dental stem cells from estrogen-deficient donors should not be used as the preferred candidate cell for dental tissue engineering. Further studies are required to explore the potential correlation between estrogen deficiency and tooth abrasion as well as the inherent mechanisms embedded in estrogen-mediated dentinogenesis of human teeth.

## Abbreviations

AB: Alveolar bone
ALP: Alkaline phosphatase
CPC: Cetylpyridinium chloride
DSP: Dentin sialoprotein
H&E: Hematoxylin and eosin
IHC: Immunohistochemistry
IOD: Integral optical density
MOD: Mean optical density
OCN: Osteocalcin
OSX: Osterix
OVX: Overariectomy
PMSF: Phenylmethylsulfonyl fluoride
RUNX2: Runt-related transcription factor 2
SABC: Streptavidin–biotin complex
SD: Standard deviation
